# Role of Hierarchical Protrusions in Water Repellent Superhydrophobic PTFE Surface Produced by Low Energy Ion Beam Irradiation

**DOI:** 10.1038/s41598-019-45132-z

**Published:** 2019-06-17

**Authors:** Vivek Pachchigar, Mukesh Ranjan, Subroto Mukherjee

**Affiliations:** 1 Institute for Plasma Research, Gandhinagar, 38248 India; 2 Homi Bhabha National Institute, Mumbai, 400094 India

**Keywords:** Structural properties, Surface patterning, Polymers, Wetting

## Abstract

The surface wettability of polytetrafluoroethylene (PTFE) was investigated with low energy Ar^+^ ion beam irradiation varied from 300 eV to 800 eV both at normal and oblique angle of incidence (0°–70°) and at a low irradiation time of few 10 s of seconds. A remarkable change in surface wettability was observed, surface became hydrophobic to superhydrophobic just at 800 eV energy and in 30 s time. A systematic increase in the contact angle was observed with increase in beam energy and irradiation time. For a given ion energy and a threshold irradiation time, the hierarchical protrusions developed that leads to the rolling and bouncing of water droplet even on the horizontal PTFE surface. For the above energy range, the rolling speed was found to be in the range of ~19–31 mm/s. This induced wetting behaviour due to ion irradiation leads to the Cassie-Baxter state as confirmed by the calculation of sliding angle, contact angle hysteresis (CAH) and surface free energy (*S*_*E*_). The CAH values were found to be reduced from 18° for untreated surface (*S*_*E*_ ~ 20 mN/m) to 2° for 800 eV, 180 s irradiated surface (*S*_*E*_ ~ 0.35 mN/m) at normal incidence.

## Introduction

Over the last two decades, fabrication of hydrophobic and superhydrophobic surfaces has extensively increased due to their superior water repellent property^1^. Also, development of functional surfaces with self-cleaning^2,3^, anti-scratch^3,4^, anti-icing^5,6^, anti-corrosion^7–9^ and fog harvesting^10^ properties has turn out to be an emerging field of research and technological applications. Superhydrophobic surfaces also have important role in water harvesting, water condensation and heat transfer^11–13^. As superhydrophobicity is achieved by having very large contact angle (θ > 150°) with low surface energy, a substantial amount of study has been carried out in order to modify surface properties either by surface structuring or by changing surface chemistry. Various fabrication processes are available to achieve hydrophobic and superhydrophobic surfaces including coating techniques like sputter deposition^14–16^, chemical vapour deposition^17–20^ and surface structuring techniques like plasma etching^21–24^, ion beam irradiation^25–28^, etc.

Polytetrafluoroethylene (PTFE) or Teflon is widely used for the fabrication of superhydrophobic surfaces due to its low surface free energy (~20 mN/m at 20 °C). Teflon and Teflon-like coatings found number of applications in automobiles, non-stick cookware, and medical applications due to high heat resistance, excellent electrical insulation and biocompatibility^29^. As PTFE is hydrophobic in nature, many investigations have been carried out to alter its wettability by modifying the surface structures. Use of various plasma treatments like Ar^30–32^, O_2_^24,33–35^ or Ar + O_2_^23,36^ modifies the wetting behaviour of PTFE. But the plasma treatment involves many process parameters like type of plasma (DC, RF, etc.), working pressure and also requires a treatment time for several hours to achieve superhydrophobicity. H.C. Barshilia *et al*.^23^ have developed superhydrophobic PTFE surface using combination of Ar and O_2_ plasma which required 4 hours of treatment time. On other hand, ion beam irradiation technique is considered to be a universal method to produce well organized structures on semiconductor^28,37^, metal^38,39^ and polymer surfaces^40–42^. Although, a very few wettability studies have been carried out on PTFE surfaces using ion beams^43–47^. Yoon *et al*.^43^ used Ar ion bombardment on PTFE surface by varying the ion fluence from 10^15^–10^17^ ion/cm^2^ and found that contact angle is increasing with increase in ion fluence. But they have not mentioned the ion beam energy. Chen *et al*.^44^ used extremely high energies of 60 keV O^3+^ and 24 keV F^4+^ ions with very low fluences and found that contact angle is decreased. Inoue *et al*.^45^ irradiated the PTFE surface with a ion energy range of 8–30 keV for the fluence range of 3.1 × 10^16^–18.3 × 10^16^ ions/cm^2^ and found that surface become ultra-hydrophobic. Lee *et al*.^46^ used fixed low energy Ar and Oxygen ions of 1.5 keV and produced hydrophobic surfaces. A. Atta *et al*.^47^ have investigated the wettability of PTFE using 3 keV Ar^+^ ion beam and concluded that Ar^+^ ion beam irradiation has increased the wettability of PTFE and made the surface hydrophilic due to formation of hydrophilic groups on the surface. They showed that upon ion beam irradiation, PTFE surface is defluorinated which resulted in C-C bond splitting and liberation of CF_2_ bonds.

In contrast to the above results^43–47^, we have found remarkable changes in wettability (increase in hydrophobicity) of PTFE just after few seconds of Ar^+^ ion beam irradiation at energy as low as 300 eV. It was observed in most of the literature that the studies are limited to only one or two higher ion energies and fixed angle of incidence. There is no mention about temperature induced structural changes in the studies performed at very higher energies of 8–30 keV and higher fluences. Based on this review of ion beam irradiation on PTFE surface, it was found that a systematic and in depth study at lower ion energy range (300–800 eV) with different angle of incidence (0°–70°) and fluences is completely missing. Therefore, we report a systematic study which is desirable to understand the wetting behaviour of PTFE with Ar^+^ ion beams on the above mentioned parameter range.

In the present study, we have observed that PTFE surface become superhydrophobic under lower energy (300–800 eV) Ar^+^ ion beam irradiation. Also, with the help of oblique incidence irradiation surface can become superhydrophobic at much lower fluences and energies without any additional gas and high energy ion beams. The surface morphology and the surface roughness are analysed using Field Emission Scanning Electron Microscopy (FESEM) and Atomic Force Microscopy (AFM), respectively. The performance of superhydrophobic surface is investigated by contact angle, surface free energy and rolling speed calculation. We also demonstrate the transition from Wenzel^48^ to Cassie-Baxter^49^ state of PTFE surface after higher beam energy and longer irradiation time with the help of optical images of water drop, contact angle hysteresis, sliding angle and surface free energy measurements. For technological view point, this technique would be helpful to develop superhydrophobic bulk PTFE sheets in very less time duration and specific surface region can be make superhydrophobic by using masking or ion beam writing.

## Results and Discussion

### Surface morphology investigation by FESEM

**Figure 1:**
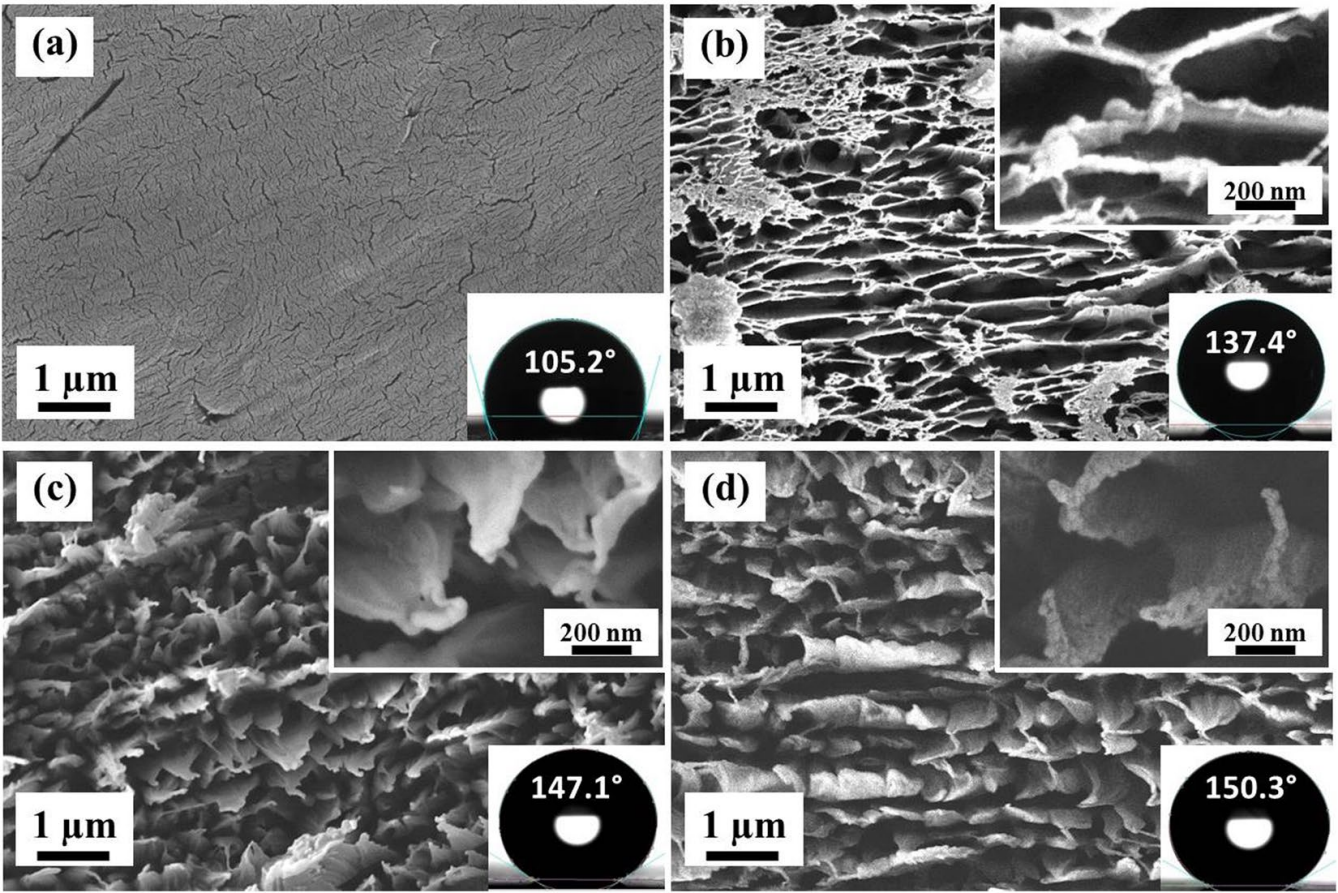
FESEM images of pristine and ion beam treated PTFE surfaces at normal angle of incidence. (**a**) Pristine, (**b**) 300 eV, (**c**) 500 eV and (**d**) 800 eV. High magnification images are shown in insets. Ion beam treatment time is 60 s.

Figure 1 shows the surface morphology of pristine as well as ion beam treated PTFE surfaces at different ion energies for same time duration of 60 s. The pristine sample looks very smooth with several cracks on its surface (Fig. 1a). At 300 eV, irregular structures with long cross chains (Fig. 1b) are observed. At 500 eV, the cross linked chains break and regular sub-micron scale structures start forming with sharp top edges as shown in the inset view of Fig. 1c. Further increase in the beam energy results in more pronounced and sharp structures. The hierarchical structures have been observed with nano-scale roughness on micro-scale structures (Inset view of Fig. 1d). At lower beam energy of 300 eV and 60 s irradiation time, the Ar^+^ ions energy is not high enough to create separated microstructures on the surface and only long cross-chains are produced on the surface, reflecting the initiation of structure formation. Penetration depth of Ar^+^ ions in PTFE would be higher in case of 800 eV as compare to 300 eV, hence, Ar^+^ ions penetrate deep inside the surface, break the chains and produce sharper edged microstructures with increasing in ion beam energy.

**Figure 2:**
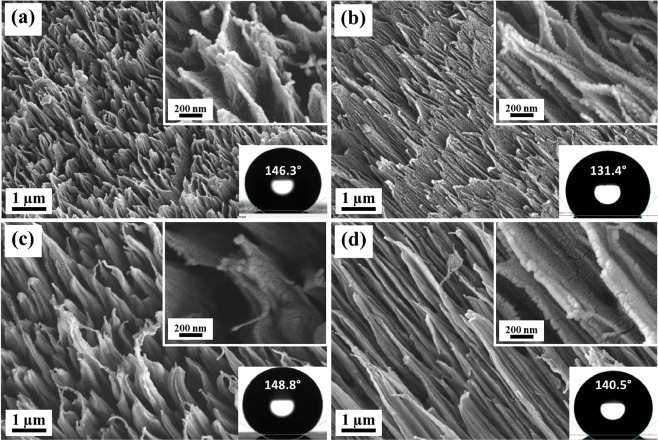
FESEM images of ion beam treated PTFE samples at beam energy and angle of incidence of (**a**) 300 eV, 40°, (**b**) 300 eV, 70°, (**c**) 800 eV, 40° and (**d**) 800 eV, 70°. High magnification images are shown in insets. Ion beam treatment time is 60 s.

Figure 2 shows the surface morphology of PTFE, ion irradiated at oblique angle of incidence (40° and 70°) with respect to the surface normal for same time duration of 60 s. In this case, well aligned regular structures are observed in the direction of ion beam even at beam energy of 300 eV and angle of incidence of 40° (Fig. 2a). As the angle of incidence is increased to 70°, the structures are stretched in the direction to incident beam (Fig. 2b). The same result is observed at 800 eV with larger and more stretched structures (Fig. 2b,c). In case of oblique angle of incidence irradiation, hierarchical structures are observed.

**Figure 3:**
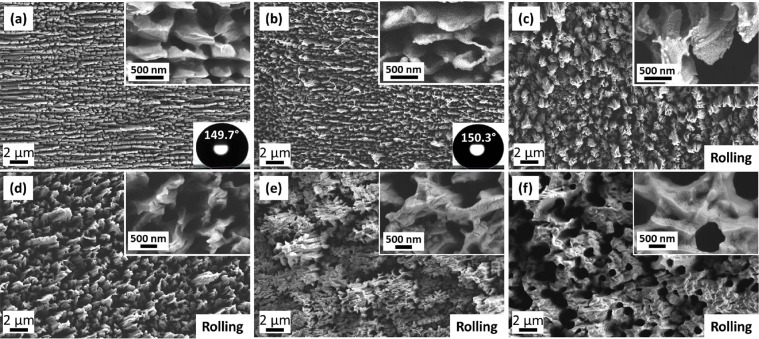
FESEM images of ion beam treated PTFE surfaces at 800 eV and normal angle of incidence. The treatment time is (**a**) 30 s (**b**) 60 s (**c**) 180 s (**d**) 240 s (**e**) 540 s and (**f**) 780 s.

Figure 3 shows the role of irradiation time (ion fluence) on the morphology of irradiated PTFE surfaces for 800 eV beam energy. Initially, at low time duration, long cross-chains are observed (Fig. 3a), which are then converted into micro-structures (Fig. 3b). As irradiation time is increased further, the micro-structures become larger in size (Fig. 3c–e) and finally a porous surface is developed at 780 s with pore size between 0.5–1.0 µm (Fig. 3f).

### Surface roughness analysis by AFM

**Figure 4:**
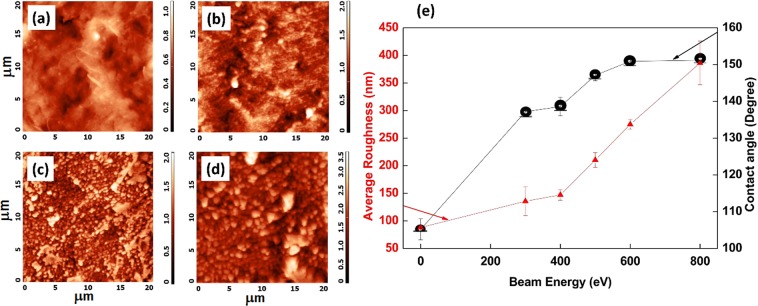
AFM images and average roughness investigation of pristine and ion beam treated PTFE surfaces at normal angle of incidence. (**a**) Pristine, (**b**) 300 eV, (**c**) 500 eV and (**d**) 800 eV. All the scales are in *µm*. (**e**) Variation in average roughness and contact angle of PTFE surface with ion beam energy for normal angle of incidence of ion beam. Ion beam treatment time is 60 s.

Figure 4 shows the AFM topography of PTFE surface and variation in the average surface roughness (*R*_*a*_) with ion beam energy from 300 eV to 800 eV for 60 s at normal incidence. Pristine PTFE is very smooth (Fig. 4a) with *R*_*a*_ equals to 88.0 nm. Upon irradiation at 300 eV, irregular features start forming on the surface and therefore *R*_*a*_ increases to 135.0 nm. A drastic increase in surface roughness is observed after irradiation with 400 eV beam energy (*R*_*a*_ ~ 147 nm) and reaches to the maximum value of 386.3 nm for 800 eV. A systematic increase in the contact angle is clearly seen with increase in surface roughness. But, in spite of increase in *R*_*a*_, saturation in the contact angle is observed after irradiation with 400 eV beam energy. This systematic increase in surface roughness is a result of increase in surface structures size with increase in beam energy, which can be further confirmed by FESEM images.

### Contact angle measurement

**Figure 5:**
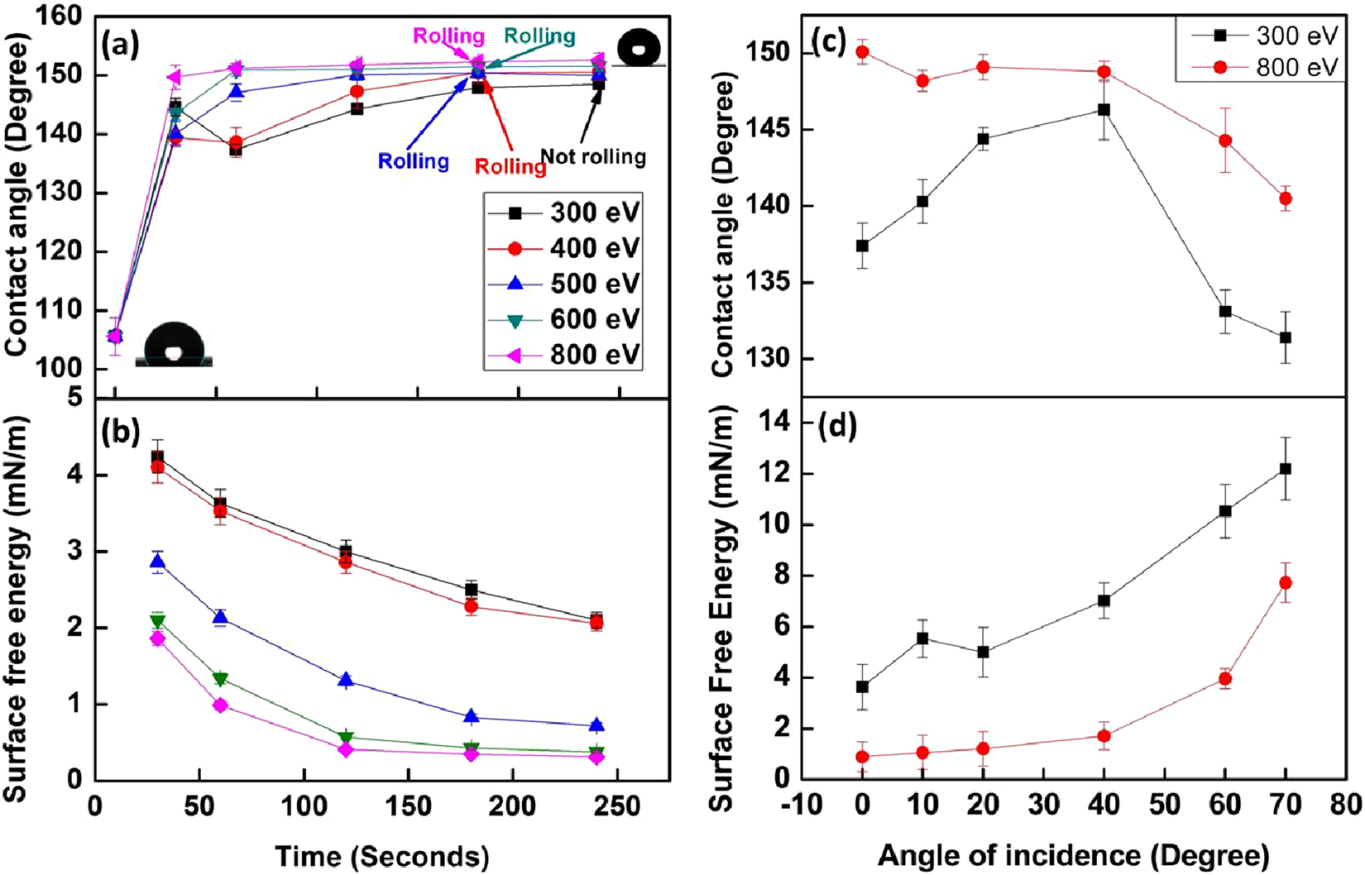
Contact angle and surface energy measurements of ion beam irradiated PTFE **(a)** Change in contact angle with beam energy and treatment time of the PTFE surface irradiated at normal angle of incidence. (**b**) Variation in surface free energy of PTFE surface with change in beam energy and ion beam treatment time at normal angle of incidence. (**c**) Change in contact angle with angle of incidence of ion beam for 300 eV and 800 eV energies, respectively. (**d**) Variation in surface free energy of PTFE surface with change in angle of incidence of ion beam for beam energy of 300 eV and 800 eV. Ion beam treatment time is 60 s.

Since, the contact angle investigation has turned out to be a simple approach to study the wettability of any surface, water contact angle (WCA) measurement was carried out using a sessile drop method. Figures 1–3 also show the value of contact angle in inset of every image. As shown in Fig. 1a, Untreated PTFE is already hydrophobic (WCA: 105.2°) in nature. Figure 5a shows the variation in the contact angle with ion beam energy and irradiation time. After irradiation for 30 s only, the contact angle has reached a range between 140°–150° corresponding to its beam energy. At lower irradiation time, i.e. 30 s and 60 s, scattered contact angle values are observed with increase in beam energy, but, after 120 s, a systematic increase in contact angle has been observed with increase in beam energy. In case of 800 eV, the contact angle of PTFE surface reaches to 149.7° for 30 s and increases to a maximum value of 152.6° for 240 s with very less deviation. Figure 5a also shows the threshold value of irradiation time for which the water droplet starts rolling over the horizontal surface corresponding to each beam energy. Above the threshold irradiation time, adhesion of water droplet with PTFE surface becomes so low that the water droplet just rolls off when it is dispensed even on a horizontal surface (Movie S1). It can be observed that water droplet does not roll even after 240 s of irradiation time for 300 eV, however, as the beam energy increases, droplet starts rolling at just 180 s of irradiation time for 400 to 800 eV energy and at normal incidence irradiation. Although the threshold irradiation time is same for above mentioned energies (400–800 eV), the speed at which water droplet rolls from the surface is quite different for different energies.

Table 1 shows the measurement of rolling speed of water droplet as a function of ion beam energy and irradiation time. It also represents the threshold value of time duration for a water droplet to roll off from a surface. The rolling phenomena for 300 eV irradiation are observed only after irradiation of 600 s with rolling speed of 19.1 mm/s. As the beam energy increases, the rolling speed also increases and reaches to 31.2 mm/s for 800 eV. As the beam energy is increased, the ion beam penetration in the surface also increases and therefore the size of structures became larger at 800 eV. Since, the air gap between the structures in case of 800 eV irradiation is much higher than 400 eV, the upward force on water droplet during rolling will be higher, which in turn increases the rolling speed of the water droplet at higher beam energies.

**Table 1:** Measurement of rolling speed of water droplet from horizontal PTFE surface treated at different beam energy and time duration.

Sr. No	Beam Energy (eV)	Treatment time (s)	Rolling or not	Rolling speed (mm/s)
1	300	30–540	No	—
2		600	Yes	19.1 ± 3.5
3	400	30–120	No	—
4		180	Yes	20.2 ± 2.4
5	500	30–120	No	—
6		180	Yes	20.4 ± 1.7
7	600	30–120	No	—
8		180	Yes	26.5 ± 1.4
9	800	30–120	No	—
10		180	Yes	31.2 ± 3.1
11	1000	30	No	—
12		60	Yes	30.7 ± 1.8

Figure 5c shows the variation in the contact angle with angle of incidence of ion beam. For 300 eV, as the angle of incidence is increased from 0° to 40°, there is a systematic increase in the contact angle from 137.4° to 146.3° respectively. This increment is followed by a drastic reduction in contact angle for 60° (WCA: 133.1°) and 70° (WCA: 131.4°) of angle of incidence. But, a continuous reduction (WCA: 150.1° to 140.4°) in contact angle is observed with increase in angle incidence (0° to 70°) in case of 800 eV. It is known that ion penetration depth changes with ion energy and angle of incidence. At normal angle of incidence higher energy ion will penetrate more dip inside the surface. Therefore, at higher energies of around 600 eV and above larger structures are formed with higher surface roughness. Similarly, it is also known that when angle of incidence increases penetration depth initially increases and then start reducing assuming a Gaussian distribution of energy profile inside layers. At the higher grazing angles ion impact mainly remains near the surface resulting in lower roughness and hence contact angle. These are the probable reason that since with angle of incidence initially roughness increases and then reduces, results in the observed variation in contact angle.

The wetting state of any liquid on a rough solid surface is described by two wettability models: Wenzel state^48^ and Cassie-Baxter state^49^. According to Wenzel state theory, the liquid penetrates inside the grooves of the surface. If the initial surface is hydrophobic, then the hydrophobicity is enhanced by increasing the surface roughness and the apparent contact angle (*θ*_*W*_) is given by,

1 $$cos{\theta }_{W}=rcos\theta $$

where, *r* is the roughness factor, which is defined as the ratio of the actual surface area to the projected surface area and *θ* is the contact angle on a plain surface. Whereas, Cassie-Baxter theory states that the air pockets are trapped in between the grooves of the rough surface and liquid does not penetrate through the grooves. In this case, the apparent contact angle (*θ*_*CB*_) is given by,

2 $$cos{\theta }_{CB}=fcos\theta +(f-1)$$

where, *f* is the fraction of solid-liquid interface. Therefore, when a liquid is in a Cassie-Baxter State, it exhibits a higher contact angle which is a basis for achieving the superhydrophobic surfaces. Hence, Cassie-Baxter state is attributed to a very low adhesion and high mobility of liquid droplet on a solid surface due to very less solid-liquid interaction. Since, the formation of microstructures has a great dependence on the beam energy and ion fluence, the rolling of water droplet also depends on both of these parameters. Here, *θ*_*CB*_ can be well predicted by obtaining the *f* value from FESEM image analysis and can be compared with experimentally measured values. However, it is very clear from FESEM images that ion beam irradiated PTFE surface are not very regular in shape, some places they are tilted and twisted. Based on this FESEM contrast information, it is difficult to calculate the exact or nearly exact solid area fraction (*f*) with the available image analysis software (ImageJ). In case of angle of incident even structures are tilted, so it is even more difficult to calculate the solid area fraction. However, an attempt has been made to calculate the value of *f* for few clean cases for normal incidence irradiation asshown in Table 2 and found that measured and calculated contact angles are nearly same with maximum error of 8.5%, due to the fact that calculated *f* is always higher than the actual values. This matching of measured and calculated contact angle clearly indicates that produced surfaces after ion beam irradiation are in Cassie-Baxter state.

**Table 2:** Calculation of solid area fraction (*f*) and respective theoretical (*θ*_*CB*_) and experimental (*θ*_*M*_) contact angle values for ion beam irradiated PTFE surface at different angle of incidence (*θ*_*i*_).

Beam Energy: 800 eV, Treatment time: 60 s				
*θ*_*i*_	*f*	*θ*_*CB*_	*θ*_*M*_	%error
0	0.19	150.5	151.2	0.46
40	0.42	136.3	148.8	8.40
70	0.37	138.9	140.5	1.13

In this study, the surface roughness and the contact angle have increased with increase in beam energy and irradiation time. For lower energy (300–500 eV) and irradiation time (30–120 s), the water droplet remains in Wenzel state, because, upon increase in surface roughness, the contact angle increases, but the adhesion of droplet remains high enough to prevent its rolling. After the threshold irradiation time for each beam energy, water droplet starts rolling due to very low adhesion of droplet with the surface and it may be described as the transition of droplet from the Wenzel state to the Cassie-Baxter state as shown above. It can also be confirmed by FESEM analysis, which shows the formation of micron size structures with large pores on PTFE after irradiation at 800 eV with longer time duration (Fig. 3). A clear transition in wetting state is seen from 180 s irradiation time, after which droplet rolls off from the surface. This wetting transition is further confirmed by contact angle hysteresis (CAH), sliding angle measurements (Table 3) and magnified optical images of water drop resting on untreated and treated surfaces (Fig. S1). The untreated PTFE surface has CAH value of 18.2°, which gradually decreases with ion beam energy and treatment time. For 300 eV, CAH decreases from 12.3° for 60 s to 3.5° for 540 s. Upon increase in beam energy to 500 eV, CAH decreases from 6.2° for 60 s to 4.3° for 180 s and finally attains the value of 1.8° for 800 eV, 180 s. Similarly, sliding angle values are also decreasing with ion beam energy and treatment time. The untreated PTFE is not rolling from the surface even after tilting the manual tilting stage upto 70° (sliding angle is beyond the instrument range). Similar results are observed for 60 s and 180 s irradiated surfaces at 300 eV beam energy, but after 540 s irradiation, sliding angle became 8°. When beam energy has increased from 500 eV to 800 eV, the sliding angle has also decreased from 28° to 3° for 60 s treatment time. The rolling phenomena is observed in both the cases for 180 s. Figure S1 shows the optical images of water drop resting on untreated and ion beam treated surface. When water drop rests on untreated surface, it fills the whole surface area which is shown by white dotted box in Fig. S1. After irradiation at 300 eV for 60 s, same result is observed, which shows that the water drop remains in the Wenzel state. But, after irradiation at 800 eV for 180 s, water droplet does not penetrate inside the structures due to large upward force provided by air pockets between the structures, which are clear indication of Cassie-Baxter state. Theses combined results from optical images, rolling phenomena, CAH and sliding angle measurements have confirmed that low energy ion beam irradiation can tune the Wenzel and Cassie-Baxter states by tuning its beam energy and treatment time on PTFE surfaces.

**Table 3:** Variation in contact angle hysteresis and sliding angle with ion beam energy and treatment time.

Sr. No	Beam Energy (eV)	Treatment time (s)	Contact angle hysteresis (Degree)	Sliding angle(Degree)
1	Untreated		18.2°	—
2	300	60	12.3°	—
3		180	10.3°	—
4		540	3.5°	8°
5	500	60	6.2°	28°
6		180	4.3°	Rolling
7	800	60	4.2°	3°
8		180	1.8°	Rolling

### Surface free energy

The surface free energy (*S*_*E*_) of irradiated PTFE surfaces is calculated using OWRK^50^ method as described elsewhere^47,50^, which involves determination of surface free energy by measuring contact angle of a solid surface by a polar (e.g. water) and a non-polar (e.g. diiodomethane) liquid with known polar (${\gamma }_{lv}^{P}$) and non-polar (${\gamma }_{lv}^{D}$) components as shown in Table S1. As the surface free energy of any solid surface is the sum of its polar (${\gamma }_{sv}^{P}$) and non-polar (${\gamma }_{sv}^{D}$) components, it is derived from the Young’s equation as follows:

Young’s contact angle equation is given by,

3 $${\gamma }_{lv}cos\theta ={\gamma }_{sv}-{\gamma }_{sl}$$

where, *γ*_*sl*_, *γ*_*lv*_, and *γ*_*sv*_ are solid-liquid, liquid-vapor and solid-vapor interfacial tensions respectively. *γ*_*sl*_ can also be written below as proposed by OWRK model:

4 $${\gamma }_{sl}={\gamma }_{sv}+{\gamma }_{lv}-2(\sqrt{{\gamma }_{sv}^{D}{\gamma }_{lv}^{D}}+\sqrt{{\gamma }_{sv}^{P}{\gamma }_{lv}^{P}})$$

After substituting the value of ${\gamma }_{sl}$ from eq. (3) to eq. (4) and simplifying, one would get the following equation which indicates the equation of a straight line.

5 $$\sqrt{{\gamma }_{sv}^{D}}+\sqrt{{\gamma }_{sv}^{P}}\sqrt{\frac{{\gamma }_{lv}^{P}}{{\gamma }_{lv}^{D}}}=\frac{1}{2}\frac{{\gamma }_{lv}(1+cos\theta )}{\sqrt{{\gamma }_{lv}^{D}}}$$

By solving eq. (5) for $\sqrt{{\gamma }_{sv}^{P}}$ and $\sqrt{{\gamma }_{sv}^{D}}$, the polar and non-polar component of surface free energy can be determined, which in turn summed up to the total surface energy of any solid surface.

Figure 5b shows the variation in the surface free energy of irradiated PTFE surfaces at various beam energies and normal angle of incidence as function of irradiation time. For a particular beam energy, *S*_*E*_ decreases as a function of irradiation time. *S*_*E*_ starts decreasing almost linearly for lower beam energy (300 eV and 400 eV) and becomes exponentially decreasing for higher beam energy (600 eV and 800 eV). For 300 eV, *S*_*E*_ varies as 4.24 mN/m, 3.63 mN/m, 3 mN/m, 2.5 mN/m and 2.1 mN/m. In case of 800 eV, *S*_*E*_ starts from 1.86 mN/m for 30 s irradiation and reached to 0.31mN/m for 240 s irradiation time. When PTFE surface is irradiated at oblique angle of incidence, a systematic increase in surface free energy is observed with increase in the angle of incidence (Fig. 5d). For small change in angle of incidence (upto 20°), *S*_*E*_ varies irregularly and very slow for the beam energy of 300 eV and 800 eV respectively, but follows linear and exponential growth respectively thereafter. Table 4 shows the polar and dispersive parts of surface free energy of irradiated PTFE surfaces measured using OWRK method. Both ${\gamma }_{sv}^{P}$ and ${\gamma }_{sv}^{D}$ decreases with increase in beam energy and irradiation time. For the beam energies of 300 eV and 400 eV, no significant change in ${\gamma }_{sv}^{P}$ and ${\gamma }_{sv}^{D}$ is observed. With systematic increase in beam energy, polar component has gradually decreased to zero for 180 s and 240 s irradiation time. Low surface free energy leads to high mobility, large contact angle and low adhesion of water with solid surface.

**Table 4:** Dispersion and polar components of surface energy of irradiated PTFE for various beam energy and time duration.

Sr. No	Beam energy (eV)	Treatment time (s)					
		30 s		180 s		240 s	
		${{\boldsymbol{\gamma }}}_{{\boldsymbol{sv}}}^{{\boldsymbol{D}}}$ (mN/m)	${{\boldsymbol{\gamma }}}_{{\boldsymbol{sv}}}^{{\boldsymbol{P}}}$ (mN/m)	${{\boldsymbol{\gamma }}}_{{\boldsymbol{sv}}}^{{\boldsymbol{D}}}$ (mN/m)	${{\boldsymbol{\gamma }}}_{{\boldsymbol{sv}}}^{{\boldsymbol{P}}}$ (mN/m)	${{\boldsymbol{\gamma }}}_{{\boldsymbol{sv}}}^{{\boldsymbol{D}}}$ (mN/m)	${{\boldsymbol{\gamma }}}_{{\boldsymbol{sv}}}^{{\boldsymbol{P}}}$ (mN/m)
1	300	3.89	0.35	2.32	0.18	1.98	0.12
2	400	3.78	0.32	1.55	0.73	1.91	0.15
3	500	2.56	0.3	0.57	0.26	0.62	0.1
4	600	1.84	0.26	0.4	0.03	0.38	0
5	800	1.7	0.16	0.35	0	0.31	0

**Figure 6:**
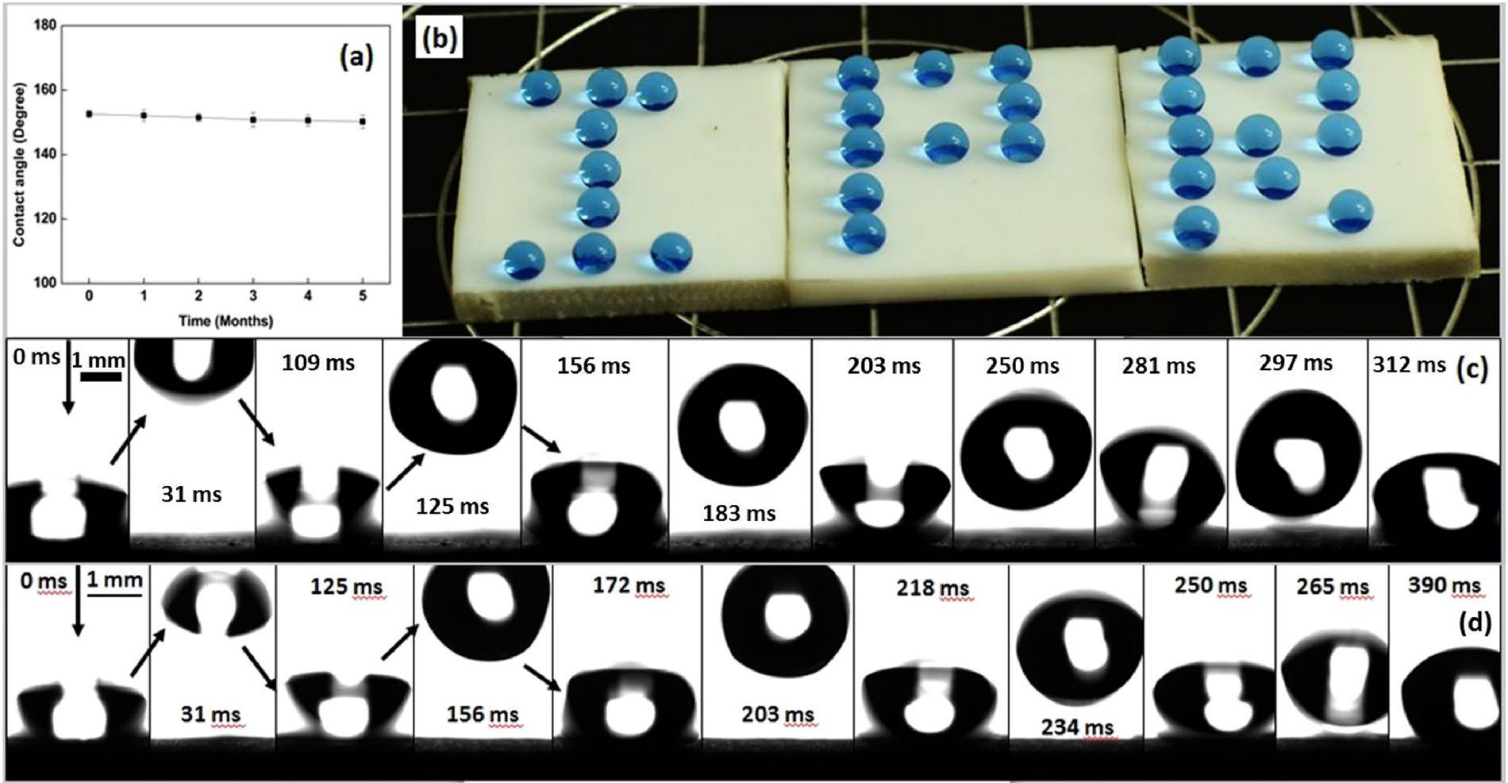
(**a**) Performance of superhydrophobic PTFE surface under ambient environment, (b) Photograph of spherical water droplets resting on superhydrophobic PTFE surface and Demonstration of bouncing effect of water droplet which is dispensed 10 mm above the surface for 800 eV and 180 s irradiation at (**c**) normal angle of incidence and (**d**) 40° with respect to surface normal.

The durability of superhydrophobic PTFE surface has been observed by keeping the sample in air medium for several months and checking the contact angle at periodic time. Figure 6a shows the performance of superhydrophobic PTFE surface in ambient environment. PTFE surface retains its superhydrophobicity even after five months with a decrease in contact angle from 152.6° to 150.2°. Figure 6b shows the photograph of the word “IPR” written with water droplets on the superhydrophobic PTFE surface achieved after ion beam treatment. A blue colour dye has been mixed with DI water in order to present the photograph effectively. The water droplet of about 10 µL in volume (radius of spherical droplet of about 1.4 mm) was used to perform the bouncing test from 10 mm above the surface. Fig. 6c,d shows the bouncing droplet on 800 eV irradiated PTFE for 180 s at normal and 40° angle of incidence, respectively. The vertical height obtained by water drop after first impact is almost same for both the cases. However, at normal incidence droplet start rolling, but at 40° angle of incidence droplet only jumps few times and then remain static in its position without rolling. As discussed earlier, the height of the structures formed in PTFE surface at oblique incidence is less, which results in lower air pockets within the structures and give relatively lesser push to the water droplets for rolling and bouncing, which prevent the water drop from rolling while bouncing.

In summary, we have observed that ion beam irradiation leads to a clear growth in structure formation with increase in beam energy and irradiation time. Increase in the surface roughness with improved structures is a result of stretching of the protrusions, not the sputtering and re-deposition of material^41,42^. Due to this phenomena, higher beam energy irradiation results in nicely separated high aspect-ratio structures, which are the basis for the formation of superhydrophobic surfaces. The air pockets created between the microstructures prevent the water droplet to spread on completely on a surface and reach to the Cassie-Baxter state. PTFE surface became so superhydrophobic that the water droplet just rolls off from the horizontal surface like “Lotus Effect”^51^. This effect is derived from the lotus leaf, in which hierarchical structures are observed. Upon ion beam irradiation, PTFE surface also exhibit excellent water repellent property with hierarchical structures on its surface.

## Conclusions

Superhydrophobic and water repellent PTFE surface is fabricated using low energy Ar^+^ ion beam irradiation. FESEM investigations reveal the formation of regular hierarchical protrusions at higher ion energy and glancing angle irradiation. A systematic increase in the contact angle has been observed with increase in ion energy and hence the surface roughness as confirmed by AFM analysis. Ion beam energy dependent contact angle study has shown the saturation in contact angle values for longer irradiation time, but, after a threshold time according to the beam energy, the water droplet starts rolling over the surface. The surface free energy reduces more rapidly in case of 600 eV and 800 eV beam energy compare to the low energy irradiation. Calculation of solid area fraction, *f* concluded that experimental *θ*_*M*_ values are well matched with theoretically calculated *θ*_*CB*_ values. Also, large reduction in sliding angle and CAH values of ion irradiated surface has further confirmed the Cassie-Baxter state of water droplet. The minimization of polar component of surface free energy after ion beam irradiation has indicated the absence of any polar group on the superhydrophobic PTFE surface.

## Methods

### Ion beam irradiation

Commercial PTFE samples (20 mm × 20 mm × 2 mm) were first rinsed with distilled water and then cleaned with acetone and isopropyl alcohol in ultrasonicator. Specimens were placed in the ultra-high cylindrical vacuum chamber (90 cm diameter) for ion beam irradiation with base pressure of 8 × 10^−8^ mbar and working pressure of 2.3 × 10^−4^ mbar. Low energy Kaufman type ion source (Sinaris 40-i, Microsystems GmbH) with beam energy from 300 eV to 800 eV was used to irradiate PTFE specimens with Ar^+^ ions. PTFE samples were irradiated with the ion fluence from 1 × 10^16^ ions/cm^2^ to 3 × 10^18^ ions/cm^2^ and time duration from 30 s to 780 s at normal as well as oblique (10°, 20°, 40°, 60° and 70°) angle of incidence.

### Surface characterization

The surface morphology of unirradiated as well as irradiated PTFE surfaces was analysed using FESEM (Carl Zeiss Merlin VP). The variation in surface roughness before and after ion beam irradiation was investigated by AFM (NT-MDT NTEGRA). The sessile drop method was used to measure the water contact angle (CA) by using a goniometer (OCA 15EC, Dataphysics), with contact angle measurement range of 0°–180° and accuracy of ± 0.1° at room temperature (25 °C). The contact angle was measured by dispensing 10 µL of deionized water droplet on the surface. The reason behind dispensing 10 µL was that after ion beam irradiation, the adhesion of water droplet with PTFE surface became so low that the small volume droplet (~5 µL) was not able to dispense from the needle. Therefore, sufficient amount of volume was needed (~10 µL) to dispense water droplet from the needle by its own weight. The rolling speed of the water droplet was calculated by taking a video of rolling droplet and measuring a distance between two positions of a droplet and the time taken between these two positions. The contact angle hysteresis (CAH) was determined by advancing and receding contact angle measurement using sessile drop needle-in method. The sliding angle was measured by using manual tilting stage (with range of 0°–70° and accuracy of ± 1°). The surface free energy (*S*_*E*_) was calculated by measuring the contact angle of two different liquids (water and diiodomethane) software using OWRK (Owens, Wendt, Rabel and Kaelble) method^50^. The durability of the irradiated surfaces was analysed by keeping the samples in the air for five months and checking the wettability on periodic time duration.

## Supplementary information

Supplymentary Information

Movie S1

## Data Availability

All the data used to support this study will be made available upon appropriate request.
