# Supplementary material for: Role of Hierarchical Protrusions in Water Repellent Superhydrophobic PTFE Surface Produced by Low Energy Ion Beam Irradiation
Source: Sci Rep. 2019 Jun 17;9:8675. doi: 10.1038/s41598-019-45132-z (PMC6572853; doi:10.1038/s41598-019-45132-z)
Supplement: Supplementary file 1 — Supplymentary Information [file 41598_2019_45132_MOESM1_ESM.docx]

**Role of Hierarchical Protrusions in Water Repellent Superhydrophobic PTFE Surface Produced by Low Energy Ion Beam Irradiation**

Vivek Pachchigar^1, 2*^, Mukesh Ranjan^1,2^ and Subroto Mukherjee^1,2^

^1^Institute for Plasma Research, Gandhinagar-38248, India

^2^Homi Bhabha National Institute, Mumbai-400094, India

*Email: vivek.pachchigar@ipr.res.in; ranjanm@ipr.res.in

**Supplementary Information**


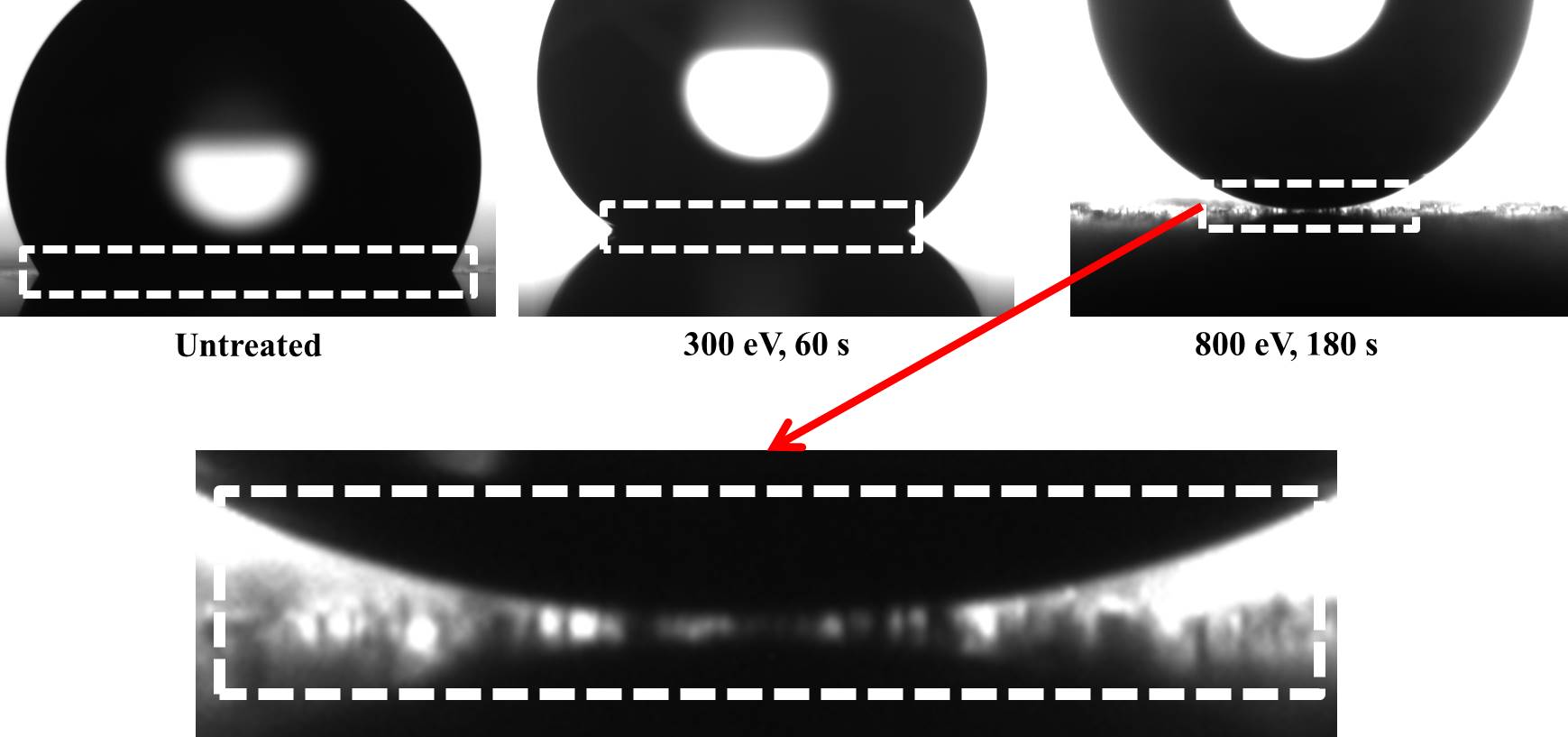


**Figure S1.** Water droplet on untreated and ion beam treated PTFE surface. White dotted box shows that the air pockets are not formed in case of untreated and 300 eV, 60 s irradiated surface. Air pockets are clearly seen in case of 800 eV, 180 s irradiated surface.

**Table S1.** Surface free energy and its components of known liquids.

| **Sr. No** | **Liquid** | $\boldsymbol{\gamma}_{\boldsymbol{lv}}$ **(mN/m)** | $\boldsymbol{\gamma}_{\boldsymbol{lv}}^{\boldsymbol{P}}$**(mN/m)** | $\boldsymbol{\gamma}_{\boldsymbol{lv}}^{\boldsymbol{D}}$ **(mN/m)** |
| --- | --- | --- | --- | --- |
| 1 | Water | 72.80 | 43.70 | 29.10 |
| 2 | Diiodomethane | 50.80 | 1.30 | 49.50 |

**Table S2.** Contact angle values for water and diiodomethane used to calculate surface energy components shown in Table 4.

| **Sr. No** | **Beam energy (eV)** | **Contact angle (Degree)** | | | | | |
| --- | --- | --- | --- | --- | --- | --- | --- |
|  |  | **30 s** | | **180 s** | | **240 s** | |
|  |  | **Water** | **Diiodo-methane** | **Water** | **Diiodo-methane** | **Water** | **Diiodo-methane** |
| 1 | 300 | 144.6 | 118.7 | 147.9 | 126.4 | 148.6 | 128.7 |
| 2 | 400 | 145.2 | 119.2 | 150.5 | 130.1 | 150.6 | 130.2 |
| 3 | 500 | 142.5 | 118.2 | 149.5 | 142.2 | 150 | 141 |
| 4 | 600 | 143.5 | 120.6 | 151.5 | 144.7 | 152.4 | 145.6 |
| 5 | 800 | 149.7 | 128.3 | 152.3 | 143.7 | 152.6 | 146.8 |
